# In Vitro Antidiabetic Effects and Antioxidant Potential of* Cassia nemophila* Pods

**DOI:** 10.1155/2018/1824790

**Published:** 2018-01-23

**Authors:** Gauhar Rehman, Muhammad Hamayun, Amjad Iqbal, Saif Ul Islam, Saba Arshad, Khair Zaman, Ayaz Ahmad, Adeeb Shehzad, Anwar Hussain, InJung Lee

**Affiliations:** ^1^Department of Zoology, Abdul Wali Khan University Mardan, Mardan, Pakistan; ^2^Department of Botany, Abdul Wali Khan University Mardan, Mardan, Pakistan; ^3^Department of Agriculture, Abdul Wali Khan University Mardan, Mardan, Pakistan; ^4^Department of Chemistry, Abdul Wali Khan University Mardan, Mardan, Pakistan; ^5^Department of Biotechnology, Abdul Wali Khan University Mardan, Mardan, Pakistan; ^6^Department of Biomedical Engineering and Sciences, SMME, National University of Science and Technology (NUST), H 12, Islamabad, Pakistan; ^7^Division of Plant Biosciences, School of Applied Biosciences, College of Agriculture & Life Science, Kyungpook National University, Daegu, Republic of Korea

## Abstract

The antidiabetic and antioxidant potential of ethanolic extract of* Cassia nemophila* pod (EECNP) was evaluated by three in vitro assays, including yeast glucose uptake assay, glucose adsorption assay, and DPPH radical scavenging activity. The result revealed that the extracts have enhanced the uptake of glucose through the plasma membrane of yeast cells. A linear increase in glucose uptake by yeast cells was noticed with gradual increase in the concentration of the test samples. Moreover, the adsorption capacity of the EECNP was directly proportional to the molar concentration of glucose. Also, the DPPH radical scavenging capacity of the extract was increased to a maximum value of 43.3% at 80 *μ*g/ml, which was then decreased to 41.9% at 100 *μ*g/ml. From the results, it was concluded that EECNP possess good antidiabetic and antioxidant properties as shown by in vitro assays.

## 1. Introduction

Diabetes mellitus is the collective name of metabolic abnormalities, primarily caused by the defect in secretion of insulin hormone by the pancreatic islets. It is chiefly manifested in the form of elevated levels of blood glucose (hyperglycemia). The reduced action of insulin on target tissues leads to a group of abnormalities, affecting the biochemistry and physiology of carbohydrate, fat, and protein [1, 2]. According to the reports of International Diabetes Foundation 2014, the estimated global prevalence of diabetes among adults is 8.3% (387 million), which will reach an estimated value of 53% (592 million) by the year 2035. Diabetes mellitus stands 5th among the diseases that can lead to death around the world. Approximately, 4.9 million deaths were recorded in 2014, and 8 per 20 died persons were of old age (≥79 years of age). In the technologically advanced countries, huge amount of sum is spent on the prevention and treatment of diabetes as well as on the discovery of new synthetic or natural drugs. In 2014, the global health management expenditures on diabetes reached USD 612 billion, which represented 11% of total worldwide healthcare expenditures [3]. Certainly, a large number of synthetic drugs have been discovered in the past, but these drugs were found to have side effects. Therefore, researchers were focusing to develop new drugs from natural sources that are safe without having any side effects. One of the recent developments in the field of natural products is the exploration of a potent plant species, such as* Cassia nemophila*. The species also known as* Senna nemophila*, silver* Cassia*, or desert* Cassia* is an evergreen shrub of about 6–8 feet high [4]. It belongs to the family Caesalpiniaceae and is native to Australia, which is domesticated to Khyber Pakhtunkhwa, Pakistan, some 20 years ago. It has bright green needle-like leaves and bearing large number of bright yellow flowers in the early spring. Brown papery seedpods follow the flowering stage, which remain on the plant till harvest. The pods can be removed by hand or by shearing, but shearing is preferred [5]. Various* Cassia* species have been known to possess a good amount of medicinal properties. Traditionally, Cassia species were used as a medicine against ringworm infections, scabies, as a laxative, purgative, in the treatment of leprosy and syphilis. Additionally, the species have been noticed to possess antimutagenic, antitumor, anti-inflammatory, hepatoprotective, antimicrobial, and antioxidant properties [6].

As mentioned earlier, diabetes mellitus has a close association with other metabolic abnormalities; one of the core abnormalities is oxidative stress. Biochemical studies have revealed an increased generation of Reactive Oxygen Species (ROS) in the cells and tissues of diabetic patients [3]. To tackle the ROS, the presence of potent antioxidant in the body of a patient is necessary because an antioxidant has the capacity of retarding or completely inhibiting the oxidation of other substances. In this regard, DPPH radical scavenging assay is one of the popular antioxidant assays, originally introduced by Marsden Blois of Stanford University in 1958. Several workers have used this method to investigate the antioxidant potential of synthetic drugs and natural products. Brand-Williams and his colleagues have introduced a modified version of Blois method in 1995, which is used as a reference by various group of researchers in recent days [7]. Similarly, indicators of the possible antidiabetic potential of a drug can be assessed through several in vitro assays, providing clues for its in vivo antidiabetic potential. Beside the antioxidant assays [8], several other indicator assays include (i) potential of glucose uptake across cell membrane such as that of yeast cells [9], adipose cells [10], or muscle cells [11]; (ii) capability of glucose adsorption [9]; (iii) inhibition of alpha amylase and alpha glucosidase enzymes [12].

## 2. Materials and Methods

### 2.1. Plant Material

The pods of* Cassia nemophila *from various parts of Khyber Pakhtunkhwa, Pakistan, were collected in the months of March till May. The specimens were kept in the herbarium at Abdul Wali Khan University, Mardan, before further processing.

### 2.2. Chemicals

Commercial baker's yeast, metronidazole, dimethyl sulfoxide (DMSO), solid DPPH, and ascorbic acid were purchased from Sigma Aldrich. Ethanol and methanol were purchased from Merck.

### 2.3. Preparation of Plant Extracts

The seeds (1 kg) from the pods of* C. nemophila* were washed, dried in the shade, and ground into a coarse powder. Ethanol was used as solvent for 8 h to obtain an ethanol soluble fraction, using a Soxhlet apparatus. The extract containing solvent was transferred to a rotary evaporator, where ethanol was evaporated under reduced pressure at 48°C. The crude extract (180 gm) was collected as a black dense paste. The paste was kept at 4°C and was used in various experiments to test it for bioactivity.

### 2.4. Determination of Glucose Uptake Capacity by Yeast Cells

This assay was performed according to the well-defined method of Cirillo [13]. Commercial baker's yeast was dissolved in distilled water to prepare 1% suspension. The suspension was kept overnight at room temperature (25°C). On the next days, yeast cells suspension was centrifuged at 4200 rpm (Microfuge® 16 Centrifuge, FX241.5P Rotor, 50/60 Hz and 220–240 V) for 5 minutes. The process was repeated by the addition of distilled water to the pallet until a clear supernatant was obtained. Exactly 10 parts of the clear supernatant fluids were mixed with 90 parts of distilled water to get a 10% v/v suspension of the yeast cells.

About 1–5 mg w/v of plant extract was mixed with dimethyl sulfoxide (DMSO) till dissolution. The mixture was then supplemented with various concentrations (5, 10, and 25 Mm) of 1 mL of glucose solution and incubated for 10 min at 37°C. To initiate the reaction, 100 *μ*L of yeast suspension was poured in the mixture of glucose and extract, vortexed, and incubated for another 60 minutes at 37°C. After incubation, the tubes were centrifuged for 5 minutes at 3800 rpm and glucose was estimated by using a spectrophotometer (UV 5100B) at 520 nm. Absorbance for the respective control was also recorded on the same wavelength. The percent increase in uptake was calculated by the formula:

(1) %  increase  in  glucose  uptake=Abs.  of  control−Abs.  of  sampleAbs.  of  control×100,

where control is the solution having all reagents except the test sample. Metronidazole was used as standard drug.

### 2.5. Glucose Adsorption Assay

The glucose adsorption capacity of the extract was determined by the method of Ou et al. [14]. Approximately, 1 gram of extract was added to 100 mL of glucose solution of five different concentrations (5, 10, 15, 20, and 30 mM). Each of these mixtures was mixed well, stirred, and incubated in a shaker water bath at 37°C for 6 hours, respectively. After incubation, the mixture was centrifuged at 4800 rpm for 20 minutes and finally the glucose content was determined in the supernatant by using glucose oxidase peroxidase diagnostic kit. The amount of bound glucose was determined by the given formula:

(2) Glucose  bound=G1−G6weight  of  sample×volume  of  sample.

Here, *G*1 represents the glucose concentration of the original solution, while *G*6 represents the glucose concentration after 6 hours.

### 2.6. Antioxidant Activity DPPH Radical Scavenging Assay

The free radical scavenging ability of the EECN extract was determined using DPPH according to the method of Burits and Bucar [15]. Freshly prepared 1 ml DPPH solution (0.004% w/v in 99% ethanol) was added to a 3 ml of sample (100 *μ*g/ml ethanol). The mixture was incubated at room temperature in the dark for 20 mins. After incubation, the mixture was vortexed and the absorbance was read at 517 nm using a spectrophotometer. Ascorbic acid was used as a reference and 99% ethanol was used as blank. The DPPH free radical scavenging activity was measured using the following formula:

(3) DPPH  radical  scavenging  activity%=B−SB×100,

where *B* is the absorbance of the blank; *S* is the absorbance of the sample extracts or standard.

### 2.7. Statistical Analysis

All the experiments were carried out in triplicate and the data was analyzed by *t*-test using Microsoft Excel 2010.

## 3. Results

### 3.1. Effect of EECNP on Glucose Uptake Capacity by Yeast Cells

**Figure 1:**
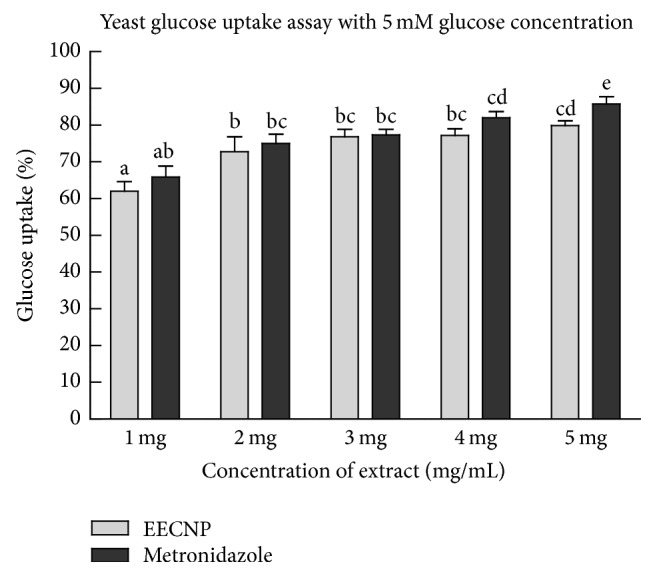
*Glucose uptake by yeast cells at 5 mM initial concentration of glucose in the presence of EECNP*. EECNP: ethanolic extract of* Cassia nemophila* pods. The error bars represent ± SE of triplicate data. The bars having different letters are significantly different at *P* = 0.05.

**Figure 2:**
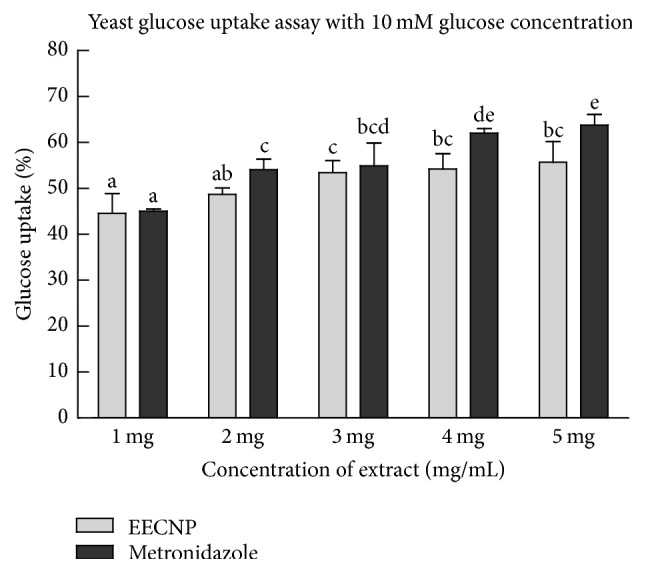
*Glucose uptake by yeast cells at 10 mM initial concentration of glucose in the presence of EECNP*. EECNP:  ethanolic extract of* Cassia nemophila* pods. The error bars represent ± SE of triplicate data. The bars having different letters are significantly different at *P* = 0.05.

**Figure 3:**
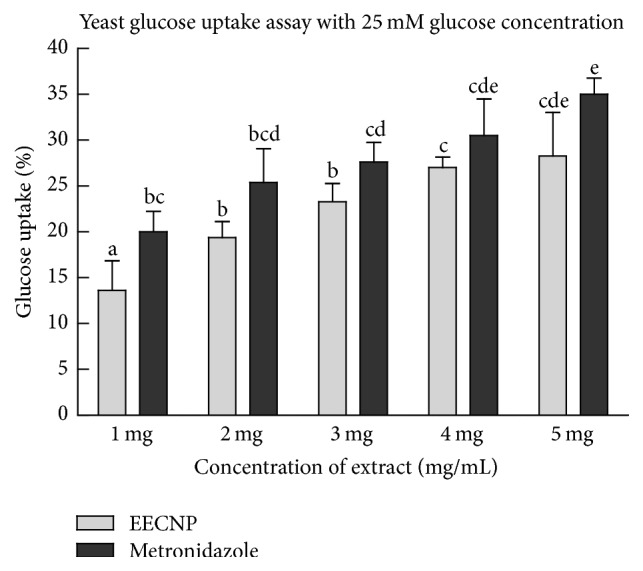
*Glucose uptake by yeast cells at 25 mM initial concentration of glucose in the presence of EECNP*. EECNP: ethanolic extract of* Cassia nemophila* pods. The error bars represent ± SE of triplicate data. The bars having different letters are significantly different at *P* = 0.05.

The ethanolic extract of* Cassia nemophila* pod promoted the uptake of glucose across the plasma membrane of yeast cells (Figures 1, 2, and 3). The glucose uptake at an initial concentration of 5 mM and 10 mM by the EECNP was comparable to that of known drug metronidazole (Figures 1 and 2). However, the effect of metronidazole on glucose uptake by the yeast cells at 25 mM glucose concentration was a bit higher as compared to that of EECNP (Figure 3). Moreover, the glucose uptake capacity at 1 mg/mL EECNP was >60% that has reached almost 80% when 5 mg/mL of EECNP was used (Figure 1). This means that by increasing the concentration of EECNP will increase the capability of yeast cells to uptake more glucose from the environment. Similarly, Figures 2 and 3 revealed a linear increase in the uptake of glucose by yeast cells with a gradual increase in the concentration of the test sample. On the other hand, an inverse relationship to the molar concentration of glucose was observed, when glucose uptake by yeast cells was compared among 5 mM, 10 mM, and 25 mM for the same amount of EECNP (Figures 1, 2, and 3). From the results it is concluded that the lower the concentration of glucose in the solution, the higher the uptake by yeast cells.

### 3.2. Effect of EECNP on Glucose Adsorption

**Figure 4:**
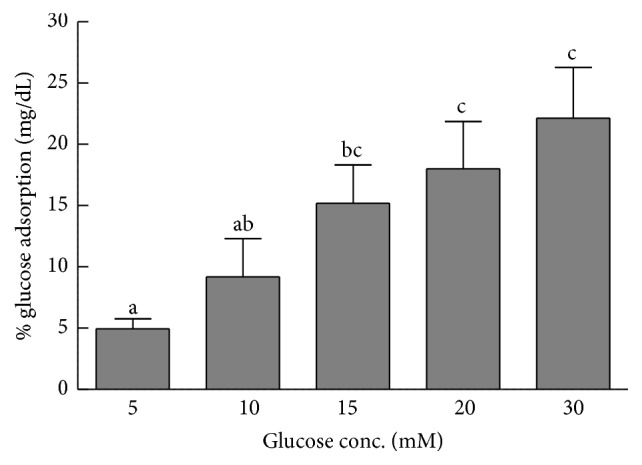
*Glucose adsorption capacity by EECNP*. EECNP: ethanolic extract of* Cassia nemophila* pods. The error bars represent ± SE of triplicate data. The bars having different letters are significantly different at *P* = 0.05.

The effect of EECNP on in vitro glucose adsorption has been shown in Figure 4. The results of the present study indicated that the extract possessed a significant glucose adsorption capacity at all tested concentrations. Moreover, the adsorption of glucose by the test sample was directly proportional to the concentration of glucose, provided that the weight of the sample was kept constant. Hence the minimum adsorption was recorded at 5 mM glucose concentration and maximum at 30 mM. Likewise, it was proved that the test extract is capable of binding the glucose even at lower concentrations.

### 3.3. Antioxidant Capacity of the EECNP

**Figure 5:**
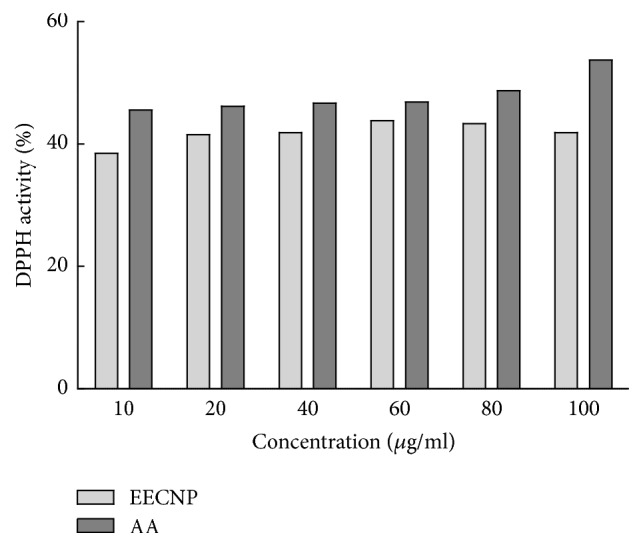
*Antioxidant activity of EECNP*. EECNP: ethanolic extract of* Cassia nemophila* pod (test sample); AA: ascorbic acid (standard).

To assess the antioxidant effect of EECP, 1,1-diphenyl-2-picrylhydrazyl (DPPH) assay was performed in vitro. As shown in Figure 5, the dose-response curve of DPPH confirms the free radical scavenging activity of the EECP. At a concentration of 25 *μ*g/ml, EECP showed maximum scavenging activity. The effect of antioxidants on DPPH is thought to be due to their hydrogen donating ability. Though, the DPPH radical scavenging potential of the extract was less than those of ascorbic acid (100%). The study showed that the extract has an average proton-donating ability and could serve as free radical inhibitors.

## 4. Discussion

The antidiabetic and antioxidant properties of ethanolic extract from the pods of* Cassia nemophila* may be attributed to the presence of bioactive compounds that might be present in* Cassia* genus [16, 17]. The previously identified bioactive compounds in* Cassia* genus include oxacyclododecan-2-one, imidazole, amentoflavone, bioflavonoids, roseanone, eugenol, caryophyllene, *β*-copaene, azulene, dodecatetraenamide, and phenethylamine [18, 19]. Therefore, the presence of one of these compounds in the extract of EECNP might help in the uptake of glucose in the present study. On a general basis, the uptake of glucose by skeletal muscles is due to the accumulation of functional glucose transporting molecules in the cell membrane. The glucose transporting molecules are regulated by leptocytes and/or myocytes in response to high secretion of insulin in blood, resulting in hypoglycemic effect [11]. On the contrary, the studies concerning the effect of drugs on reduction of postprandial hyperglycemia have been one of the important aspects in the management of diabetes mellitus, which is a well-focused therapeutic approach till date.

Furthermore, glucose uptake by yeast cells may be different from that of other eukaryotic or human body cells. Transport of glucose across yeast membrane may involve facilitated diffusion rather mediation of a phosphotransferase enzyme system or any other unknown process. The glucose uptake by the yeast cells may be affected by several variables, such as glucose concentration inside the cells or the subsequent metabolism of glucose. If most of the internal sugar is converted readily into other metabolites, the internal glucose concentration will get low and high uptake of glucose into the cell will be favored. Likewise, there are possibilities that the glucose uptake by yeast cells in the presence of EECNP extract is due to both facilitated diffusion and elevated glucose metabolism. Certainly, it will be quite interesting to explore the activity of natural extract (including EECNP) in vivo, which might help in the enhanced glucose uptake by muscle cells and adipose tissues of the body. The extract could bind glucose effectively and transport it across the cell membrane for further metabolism.

In the present study, the glucose adsorption capacity of the sample was also found to have a directly proportional relationship with the molar concentration of glucose. The adsorption property of the EECNP extract may be due to the presence of soluble and insoluble dietary fibers present in the extract. In the intestinal lumen, the adsorption of glucose by extract may possibly help in reducing the postprandial rise in blood glucose level [20]. According to Ou et al. [14], there are three possible mechanisms through which dietary fibers can help in the reduction of postprandial hyperglycemia. Firstly, they may increase the viscosity of the juices found in the small intestine and offer a hindrance in the diffusion of glucose from the lumen into the blood. Secondly, glucose may bind with these fibers lowering their concentration in the lumen of the small intestine. Thirdly, the dietary fibers may have inhibitors against alpha amylase (an enzyme that digests starch) due to which digestion of starch is inhibited reducing postprandial hyperglycemia.

Besides, the hydrogen donating ability of compounds present in the natural extracts is considered to be responsible for their antioxidant nature. Acceptance of electron by DPPH from antioxidant compounds in turn changes the colour of the solution from violet to yellow, which is measured spectrophotometrically at 517 nm [21]. Siddhuraju et al. [22] found a direct relation between antioxidant capability and the presence of phenolic contents in the crude extract of* Cassia fistula*. The elevated scavenging ability of stem, bark, and leaves of* Cassia fistula* was attributed to the presence of high concentration of tannins, proanthocyanidins, flavonols, and xanthones components. Similarly, the methanolic and ethanolic extracts of flowers of* Cassia auriculata *also possess antioxidant activity. This was demonstrated on the basis of different in vitro assays, including ABTS [23], which is in line with the observation of the current study. The pod husk of* Cassia auriculata* contains emodin, rubiadin, *β*-sitosterol, and chrysophanol [24], so there is great chances that the EECNP are also rich in such compounds.
